# The Impact of a Psychoeducational Group Program on the Mental Well-Being of Unit-Based Nurse Leaders: A Randomized Controlled Trial

**DOI:** 10.3390/ijerph20116035

**Published:** 2023-06-02

**Authors:** Amanda T. Sawyer, Hong Tao, Amanda K. Bailey

**Affiliations:** AdventHealth Research Institute, Orlando, FL 32803, USA; hong.tao@adventhealth.com (H.T.); amanda.bailey@adventhealth.com (A.K.B.)

**Keywords:** burnout, cognitive behavioral therapy, mindfulness, nurse managers, stress

## Abstract

This randomized controlled trial examined the impact of a psychoeducational group program on the mental well-being of unit-based nurse leaders, specifically nurse managers and assistant nurse managers. The program was developed around the themes of resilience, insight, self-compassion, and empowerment to fight burnout and enhance purposeful adaptive coping to reduce distress and improve mental wellbeing. The sample included 77 unit-based nurse leaders. Outcomes included post-traumatic growth, resilience, insight, self-compassion, empowerment, perceived stress, burnout, and job satisfaction. Paired samples t-tests and repeated measures ANOVA tests were conducted to compare outcomes at baseline to the follow-up timepoints of endpoint, one-month follow-up, three-month follow-up, and six-month follow-up. The intervention group participants showed significant improvement in post-traumatic growth between baseline and all follow-up timepoints compared to the waitlist control group. Among intervention group participants, there were also significant improvements in self-reflection and insight, self-compassion, psychological empowerment, and compassion satisfaction, as well as significant reductions in perceived stress, burnout, and secondary traumatic stress. This study extends existing evidence that this psychoeducational group program can be an effective intervention for improving and protecting mental wellbeing. Among nurse leaders, it can reduce stress and burnout and improve post-traumatic growth, self-reflection and insight, self-compassion, psychological empowerment, and compassion satisfaction.

## 1. Introduction

Unit-based nurse leaders have a vital role that influences patient outcomes and organizational culture [1]; however, it comes with many sources of stress. Occupational stressors are often unpredictable, urgent, and can be acute and chronic. As a result, nurse leaders are tasked with balancing multiple demands on their time and energy in high-pressure work environments. Without structural support, autonomy, and adequate rest and recovery [2,3,4], these stressors can lead to burnout which involves feelings of hopelessness, exhaustion, frustration, and difficulties in effectively doing one’s job [5]. The harmful effects of burnout in healthcare are well established. Burnout and stress in nursing management are cited as causal factors in job dissatisfaction and turnover [6,7]. Some evidence suggests that burnout among nurse managers planning to leave their positions was approximately 35% in 2018 and may be close to 75% currently [7]. Work overload, low work–life balance, competing priorities, financial responsibilities, managing staff conflicts, and lack of support contribute to the development of burnout among nursing managers [2,3,4]. Additionally, nurse managers significantly influence workplace culture and fostering a healthy work environment, with a focus on metrics, requires a tremendous amount of emotional energy and constant effort [3].

Furthermore, in the wake of the COVID-19 pandemic, unit-based nurse leaders have been responsible for providing psychosocial support to nurses while managing their own stress and exhaustion [8,9,10]. The pandemic caused extreme changes and losses that can be considered a collective trauma experienced by healthcare workers, including nurse leaders [11]. As nurse leaders are often relied upon to support direct care nurses who are exposed to trauma, this part of their job role contributes to their distress and affects their overall wellbeing [12].

While work in nursing management can put leaders at risk for these stress-related injuries, it can also provide reward, meaning, and fulfillment. Professional quality of life is a construct that encapsulates both the positive aspects of work in a helping profession (i.e., compassion satisfaction) and the negative aspect (i.e., compassion fatigue), which is divided into the two domains of burnout and secondary traumatic stress (STS) [5]. Programming that targets both symptom reduction and promotion of protective factors (i.e., compassion satisfaction) is essential for comprehensive intervention. Implicit within the programming approach should be a dual continuum understanding of mental health by attending not only to suffering but also wellbeing and thriving [13,14]. Given the impact of the pandemic, intervention should also aim to promote psychological healing and post-traumatic growth, which is a positive psychological change resulting from the process of moving through adversity, traumatic events, or crises [12,15,16,17].

While addressing burnout ultimately requires systemic changes in the work environment [18], individual-level interventions supported by organizations can buffer against acute and chronic stress and prevent escalation to more severe stress injuries (e.g., burnout, secondary traumatic stress) [16].Workplace wellbeing interventions have focused primarily on building resilience through education, though these programs have shown modest effects [19]. Previous studies have explored individual-level workplace interventions that mainly incorporate prevention and skill-based training [18,20]. Studies exploring therapeutic interventions combined with education are lacking. Moreover, much of the relevant research in hospital settings has included nurses and physicians, while the population of unit-based nurse leaders has been overlooked in the literature.

A psychoeducational group program called RISE^©^ was originally developed by a licensed mental health counselor (LMHC) in 2018 specifically for the nursing population to address the impact of stress and burnout from work as a professional caregiver [21]. The program goes beyond education to include therapeutic processing with a licensed mental health professional because those experiencing burnout and compassion fatigue might benefit from more comprehensive intervention. Bailey et al. [21] described the development and theoretical framework of the program, including operational definitions of the four themes of resilience, insight, self-compassion, and empowerment and how each is conceptualized to alleviate burnout symptoms and protect wellbeing. Given the emotional intensity of work in nursing management and the far-reaching effects of the pandemic on mental health, the program was adapted for nurse managers and assistant nurse managers in terms of content, delivery format, and intervention strategies. This adapted program called RISE for Nurse Leaders showed acceptability and feasibility in a pilot study, and participants experienced higher post-traumatic growth and empowerment [22].

The primary aim of this study is to determine whether RISE for Nurse Leaders has a significant impact on unit-based nurse leaders’ post-traumatic growth. The secondary aims are to determine whether the program affected psychological and occupational wellbeing related to resilience, insight, self-compassion, and empowerment, as well as professional quality of life, perceived stress, and job satisfaction.

## 2. Materials and Methods

### 2.1. Design

This study was a parallel randomized controlled trial with an allocation ratio of 1:1 that was conducted at a multicampus healthcare system headquartered in Florida. Participants were randomized into the intervention or waitlist control group by a computer-generated randomized number. The randomization list was saved by the principal investigator in a password-protected file, and group assignments for each participant were shared with the recruitment team member after they were enrolled in the study.

### 2.2. Sample

Using the level of significance alpha = 0.05, power = 0.8, and preliminary data from the pilot study for the primary outcome measured by the Posttraumatic Growth Inventory (mean difference = 12; standard deviation = 17) [22,23], the sample size was calculated to be 64 participants (i.e., 32 in the intervention group, and 32 in the wait-list control group). Considering an estimated attrition rate of 25 percent, an additional 8 participants were enrolled in each group. A total number of 80 participants were enrolled in the study. Recruitment occurred in January 2022. Study inclusion criteria were adult ≥ 18 years old; licensed as a registered nurse (RN); nurse manager or assistant nurse manager employed by the healthcare organization in a hospital-based setting at selected campuses in Florida; able to speak, read, and understand English fluently. The exclusion criterion in this study was employed as a direct care nurse or in another level of nursing leadership (i.e., director of nursing and executive leader).

### 2.3. Intervention

The program consists of nine weekly 90 min group sessions that fuse education through didactic content, therapeutic process through facilitation, and skill development through experiential learning and practice [21,24]. The topics of the nine weekly group sessions were previously published [22,25]. Briefly, the program sessions involve an introduction session (e.g., group guidelines, program framework, drivers, and symptoms of burnout), two resilience sessions (e.g., personal coping resources, oscillation between stress and recovery, post-traumatic growth, and connecting to purpose and meaning), two insight sessions (e.g., cognitive and emotional awareness), one self-compassion session (e.g., compassion fatigue and satisfaction, and self-compassion skills), two empowerment sessions (e.g., healthy boundaries, authentic living, and values-behavior alignment), and a closing session (e.g., synthesis of learning and self-care planning guide). Mindfulness practice serves as a foundation for learning and is infused throughout each session [21].

The program is grounded in a theoretical framework based on acceptance and commitment therapy (ACT) [26,27,28] and cognitive behavioral therapy (CBT) [29,30] with an emphasis on mindfulness [31,32]. As a multidimensional approach to improving coping and wellbeing inside and outside of the workplace, the program specifically targets the underlying causes and effects of high stress, burnout, and compassion fatigue through its four themes.

To comply with organizational policies and meet social distancing guidelines during the pandemic, the delivery of the program was adapted for this study. Synchronous groups of six to eight participants were held virtually using Microsoft Teams. The findings from the pilot study with nurse managers supported the feasibility and the benefits of a virtual synchronous group format for busy nurse leaders [22]. Additionally, while the core foundations and themes of the original program remained the same during the adaptation for unit-based nurse leaders, authentic leadership principles were integrated into the curriculum [33,34]. Authentic leadership concepts, such as relational transparency, self-awareness, values-behavior alignment, and psychological flexibility, align with the components of ACT, CBT, and mindfulness. Post-traumatic growth was added as a conceptual underpinning and informed the content, facilitation methods, and group process [22].

The intervention period was March through May 2022 for the intervention group and September through November 2022 for the waitlist control group. Participants were permitted up to three absences. Those who missed more than three sessions could continue to attend the groups but were not involved in follow-up data collection.

### 2.4. Data Collection

The following nine validated instruments were used to measure study outcomes: Posttraumatic Growth Inventory (PTGI) [23]; Professional Quality of Life (ProQOL) Scale [5,35]; Brief Resilience Scale (BRS) [36]; Self-Reflection and Insight Scale (SRIS) [37]; Self-Compassion Scale-Short Form (SCS-SF) [38]; Psychological Empowerment Instrument (PEI) [39]; General Self-Efficacy Scale (GSE) [40]; Perceived Stress Scale (PSS) [41]; Brief Index of Affective Job Satisfaction (BIAJS) [42]. Sawyer and colleagues [22] described these instruments in detail. In addition, demographic questionnaires were collected. Data collection occurred through an electronic data capture system called OpenClinica at the following timepoints: baseline in March 2022, endpoint in June 2022, one-month follow-up in July 2022, three-month follow-up in September 2022, and six-month follow-up in December 2022. Compensation was provided to participants upon completion after the final timepoint.

### 2.5. Data Analysis

Paired-samples t-tests were conducted to compare outcomes in each group between baseline and endpoint. Repeated-measures ANOVA tests were also conducted to compare outcomes longitudinally between the intervention group and waitlist control group at baseline, endpoint, one-month follow-up, and three-month follow-up. Data from the final data collection timepoint were not included in the ANOVA tests because the wait-list control group attended the program between the three-month follow-up and six-month follow-up timepoints.

### 2.6. Ethical Considerations

The study was approved by the Institutional Review Board of AdventHealth (IRBNet #1839775) and registered on ClinicalTrials.gov (NCT05254600). Informed consent was obtained from all subjects involved in the study, and they also completed pre-group screening meetings with the LMHC facilitator, which involved a traditional consent process for therapeutic services [21]. The facilitator possessed both technological and clinical competence to deliver online psychoeducation as outlined in the American Counseling Association’s Code of Ethics [43] for distance counseling. The platform used for intervention delivery, Microsoft Teams, is HIPAA compliant. Participants were informed of confidentiality limitations before consenting to the study, and the facilitator provided guidelines for online group participation to safeguard privacy (e.g., ensuring they were in a private space before logging on to the session).

## 3. Results

**Figure 1:**
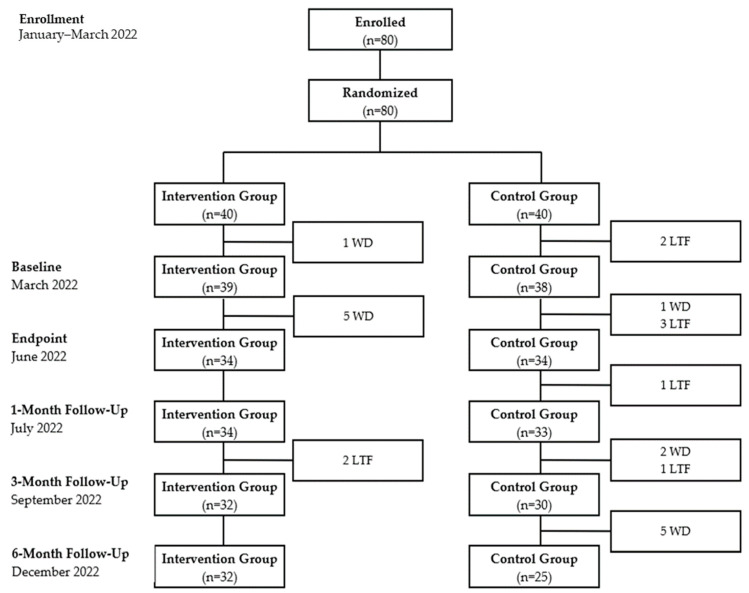
Flowchart of Participants Withdrawal (WD) (i.e., scheduling conflict, more than three absences); Lost to Follow-up (LTF) (i.e., survey non-completion).

A total of 80 nurse leaders enrolled in this study. Figure 1 shows a flowchart of participants. In the intervention group, there were the following numbers of active participants at the baseline, endpoint, one-month follow-up, three-month follow-up, and six-month follow-up timepoints, respectively: 39, 34, 32, and 32. In the waitlist control group, there were the following numbers of active participants at each timepoint, respectively: 38, 34, 33, 30, and 25. At baseline, the sample size included 30 nurse managers and 47 assistant nurse managers.

Table 1 shows the summary of the participants’ demographics. No statistically significant differences in demographic characteristics existed between the intervention and control groups. In the intervention group, 36 of the 39 participants attended the program with 83 percent completing at least six of nine sessions. In the waitlist control group, 30 participants began the program with 77 percent completing at least six of nine sessions. The results of the paired-samples t-tests comparing outcomes at baseline and endpoint are shown in Table 2, while comparisons at the one-month, three-month, and six-month follow-up timepoints are shown in Table 3. An effect size of 0.20 indicates a small effect, 0.50 indicates a medium effect, and 0.80 indicates a large effect [44]. The intervention had a small to moderate effect on many of the outcome variables.

**Figure 2:**
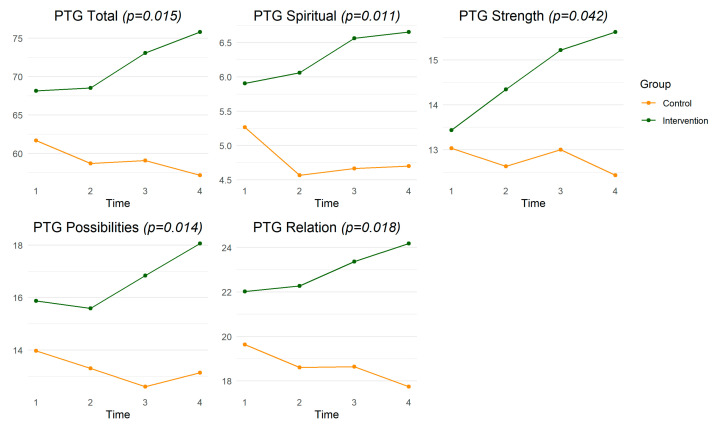
Longitudinal Between-Groups Comparison of Post-Traumatic Growth.

The results of the repeated-measures ANOVA are shown in Table 4 and Figure 2. In the figure, time 1 is baseline, time 2 is endpoint, time 3 is one-month follow-up, and time 4 is three-month follow-up.

### 3.1. Post-Traumatic Growth

Over the four timepoints of baseline, endpoint, one-month follow-up, and three-month follow-up, there were significant differences between the intervention group and control group in mean scores on the PTGI (*p* = 0.012) and its subdomains of Relating to Others (*p* = 0.018), New Possibilities (*p* = 0.014), Personal Strength (*p* = 0.042), and Spiritual Change (*p* = 0.011). Among the intervention group participants, the overall mean score on the PTGI (68.16 vs. 75.81, t = −2.72, *p* = 0.011) and the mean score in the New Possibilities subdomain (15.88 vs. 18.06, t = −2.95, *p* = 0.006) was significantly higher at three-month follow-up compared to baseline.

### 3.2. Professional Quality of Life

Among intervention group participants, there were significant improvements in the Compassion Satisfaction subdomain mean score of the ProQOL between baseline and endpoint (40.91 vs. 42.82, t = −3.07, *p* = 0.004), as well as the Burnout subdomain mean score (23.06 vs. 21.47, t = 2.30, *p* = 0.028). There was no significant difference among intervention group participants in the mean score in the STS subdomain at these timepoints (24.76 vs. 23.76). At one-month follow-up, the mean score in the Compassion Satisfaction subdomain remained significantly higher (40.91 vs. 42.38, t = −2.22, *p* = 0.033), and the mean score in the Burnout subdomain remained significantly lower (23.06 vs. 20.97, t = 3.10, *p* = 0.004). Moreover, the mean score in the STS subdomain was significantly lower at this timepoint compared to baseline (24.76 vs. 22.91, t = 2.24, *p* = 0.032).

### 3.3. Program Themes

#### 3.3.1. Resilience

There were no significant differences in the mean score on the BRS among the intervention group participants between baseline and endpoint (3.81 vs. 3.79).

#### 3.3.2. Insight

Among intervention group participants, the overall mean score on the SRIS was significantly higher at the endpoint than at baseline (89.88 vs. 93.85, t = −2.09, *p* = 0.045). The mean scores of the Insight subdomain were significantly higher at one-month follow-up (35.82 vs. 38.09, t = −2.11, *p* = 0.043) and three-month follow-up (35.53 vs. 37.78, t = −2.05, *p* = 0.048) compared to baseline.

#### 3.3.3. Self-Compassion

There were no significant differences in the mean score on the SCS-SF among the intervention group participants between baseline and endpoint (3.19 vs. 3.35). However, the mean score was significantly higher at one-month follow-up (3.19 vs. 3.42, t = −2.82, *p* = 0.008), three-month follow-up (3.18 vs. 3.44, t = −3.81, *p* = 0.001), and six-month follow-up compared to baseline (3.18 vs. 3.48, t = −3.06, *p* = 0.005).

#### 3.3.4. Empowerment

Among intervention group participants, the overall mean score on the PEI was significantly higher at the endpoint than at baseline (5.41 vs. 5.82, t = −2.86, *p* = 0.007). Mean scores in three subdomains were significantly higher after the intervention: Meaning (5.82 vs. 6.20, t = −2.37, *p* = 0.024), Competence (5.35 vs. 5.97, t = −4.90, *p* < 0.001), and Self-Determination (5.30 vs. 5.63, t = −2.08, *p* = 0.045). There was no significant difference among intervention group participants in the mean score in the domain of Impact (5.22 vs. 5.49).

Although the mean score in the Impact subdomain was not significantly higher at the endpoint, it was at the one-month follow-up (5.22 vs. 5.73, t = −2.91, *p* = 0.006). The overall mean score of the PEI and the mean score in the Self-Determination subdomain remained significantly higher at one-month follow-up (5.41 vs. 5.85, t = −3.84, *p* = 0.001; 5.26 vs. 5.69, t = −2.99, *p* = 0.005) and three-month follow-up (5.51 vs. 5.79, t = −2.09, *p* = 0.045; 5.34 vs. 5.78, t = −2.48, *p* = 0.019). The mean score in the Competence subdomain remained significantly higher through one-month follow-up (5.35 vs. 5.88, t = −4.52, *p* < 0.001), three-month follow-up (5.44 vs. 5.79, t = −2.42, *p* = 0.022), and six-month follow-up (5.44 vs. 5.90, t = −3.11, *p* = 0.004). The mean score in the Meaning subdomain remained significantly higher at the one-month follow-up (5.82 vs. 6.11, t = −2.29, *p* = 0.029), but not three-month follow-up (5.93 vs. 6.10) and again at six-month follow-up (5.93 vs. 6.23, t = −2.17, *p* = 0.038).

### 3.4. Perceived Stress, Job Satisfaction, and Self-Efficacy

Between baseline and endpoint, there were no significant differences in the mean score on the PSS (16.62 vs. 15.38), BIAJS (4.13 vs. 4.20) or GSE (33.29 vs. 33.91) among the intervention group participants. However, participants scored significantly lower on the PSS at the one-month follow-up compared to baseline (16.62 vs. 14.74, t = 2.06, *p* = 0.048).

## 4. Discussion

Considering unit-based nurse leaders’ scope of influence on patient outcomes, nurse job satisfaction, and organizational culture [1,45,46], there is scant research on interventions that support their own mental health and professional wellbeing. Moreover, studies exploring therapeutic interventions combined with education are lacking. Previous studies have explored workplace interventions that mainly incorporate education and skill-based training related to resilience and stress management or examined existing evidence-based programs (e.g., mindfulness-based stress reduction [MBSR]) adapted for specific clinical workforce populations [20,47].

This study provides evidence of the effectiveness of a psychoeducational group program designed to address the wellbeing of this essential but often overlooked population. After the program, intervention group participants showed significant improvements in post-traumatic growth, self-reflection and insight, self-compassion, psychological empowerment, and compassion satisfaction, as well as significant reductions in perceived stress, burnout, and STS. Although no significant differences in resilience, self-efficacy, or job satisfaction were found, these scores were maintained throughout the study period. There were no meaningful significant differences in outcomes between nurse managers and assistant nurse managers. This study further supports the effectiveness of the program as an intervention developed specifically for the nursing population by addressing complex experiences unique to their work.

### 4.1. Post-Traumatic Growth

The finding of higher post-traumatic growth aligns with the results of the pilot study, which was conducted during the COVID-19 Delta surge in Florida [22]. It is important to provide programming for nurses and nurse leaders during crises to facilitate such growth and to prevent an escalation of psychological distress. This program facilitates growth through active coping, cognitive processing, sharing emotions, and social support [17,48,49]. Participants told their stories, engaged in meaningful and directed self-reflection, and self-disclosed in a safe environment. Therefore, the evidence suggests the intervention can be an effective strategy to facilitate post-traumatic growth among unit-based nurse leaders, and the strategies utilized align with existing research on the drivers of growth.

Although there is limited literature about post-traumatic growth and related interventions among nurse leaders, studies examining this phenomenon among direct care nurses emerged during the pandemic. Nurses at the frontlines of the pandemic generally reported high post-traumatic growth, which was facilitated by self-disclosure, deliberate rumination (i.e., ongoing cognitive processing), self-efficacy, social support, and psychological intervention [50,51,52,53]. Recent studies show that these nurses have experienced post-traumatic growth by reflecting on their lives after experiencing struggle and positively adjusting their self-perception, interpersonal relationships, and attitude toward life [50,53]. This coping process of reflecting and constructing meaning alleviated the negative effects of nurses’ traumatic experiences related to work and helped improve satisfaction [23,53]. Strategies derived from the program’s theoretical framework facilitated this process (e.g., reappraisal, emotional awareness and expression, contact with the present, perspective taking, and values-based goal setting).

Furthermore, cognitive processing and emotional regulation can be fostered through self-disclosure of internal experiences with others in a safe, trusting environment, which is strengthened when the support network has gone through similar adversity and has knowledge of the trauma experience [49]. The venting of painful emotions and processing of internal experiences with others who have in-depth knowledge maximizes post-traumatic growth [49]. This is consistent with results from the pilot study with nurse managers in which peer support received during the program was identified as a beneficial outcome [22]. This peer support offered relief from isolation provided reassurance of shared experiences and validation and enabled learning from multiple perspectives, which contributed to growth.

### 4.2. Professional Quality of Life

As previously mentioned, professional quality of life encompasses compassion satisfaction, burnout, and STS. A study conducted before the COVID-19 pandemic found that nurse leaders have average scores in these three subdomains [54]. Nurse managers have reported higher burnout and STS than nurse executives, while nurse executives have reported higher compassion satisfaction than nurse managers [54,55]. Evidence regarding well-designed interventions to improve the professional quality of life in unit-based nurse leaders is lacking, and the present study contributes to the body of knowledge in this area.

The mean Compassion Satisfaction score increased between baseline (40.91), endpoint (42.82), and one-month follow-up (42.38) among intervention group participants. A “moderate” score in this subdomain is between 23 and 41, while a “high” score is 42 or more. Although unit-based nurse leaders dealt with significant stressors and changes during the pandemic, they also found fulfilling work experiences and outcomes related to interpersonal connection and learning. They reported relating to their staff in new ways, receiving positive feedback from staff, improved communication and delegation skills, and witnessing rewarding patient recoveries [10]. The reward and fulfillment derived from helping patients and staff and feeling competent in one’s job role contribute to compassion satisfaction, which is considered protective against fatigue and burnout [56,57]. While work environment and manager support have been associated with compassion satisfaction in nurses [58,59], results from the present study suggest that this type of individual-level intervention can also improve compassion satisfaction in nurse leaders, which, in turn, may positively impact the work environment.

The mean Burnout score on the ProQOL decreased between baseline (23.06), endpoint (21.47), and one-month follow-up (20.97) among intervention group participants. A “low” score in this subdomain is 22 or less, while a “moderate” score in this subdomain is between 23 and 41. Although it is well-established that burnout is caused mostly by environmental factors in the workplace [60], this program emphasizes coping and healing from the emotional and psychological impact of burnout, while recognizing changes in the work environment may be out of one’s control [21]. In support of the theoretical framework of the program, these findings add to the existing evidence of how mindfulness-based and CBT-based interventions can significantly alleviate burnout symptoms [61,62].

The unsustainability of the reduction in burnout scores is consistent with the literature on individual-level interventions for burnout [18,20,63], suggesting that these programs are necessary but not sufficient to comprehensively address the causes and consequences of burnout. Unit-level structural changes may lead to greater and more sustained improvements among unit-based leaders when combined with individual-level wellbeing programs. Offering an individual-level intervention, such as RISE for Nurse Leaders, in conjunction with environmental changes such as ensuring adequate staffing, supervisory support, and efficient workflow [3,64] demonstrates an organizational commitment to both nurse leader wellbeing and improvement in organizational culture. Failure to incorporate unit- and organizational-level changes may lead to cynicism, disempowerment, and mistrust among the nursing workforce [25]. The mutual commitment to improving wellbeing and reducing environmental stressors may enable improvements, especially for symptoms of burnout, to be sustained after the program.

Furthermore, hospital systems can employ chief wellness officers (CWOs) to focus on the quintuple aim, particularly the fourth aim of clinician wellbeing [65,66]. This executive leader advocates for and promotes the mental health and professional satisfaction of the healthcare workforce [60]. They are also responsible for overseeing assessment, intervention development, evaluation, quality improvement, and community building around positive workplace culture and individual mental health [60]. Following the lead of the CWO, the organization can provide staffing and resources to support programs such as RISE for Nurse Leaders.

Although the mean STS score did not change between baseline (24.76) and endpoint (23.76) and remained in the “moderate” range between 23 and 41, it did decrease to 22.91 at the one-month follow-up. Participants in the program received education about the causal factors and symptoms of traumatic stress, as well as skills to cope with severely distressing reactions. Experiential processing allowed participants to approach, rather than avoid, distressing internal experiences (i.e., thoughts, feelings, memories) and use self-compassion, emotion regulation skills, and interpersonal feedback to accept these experiences without being overcome by them [48]. Methods to facilitate post-traumatic growth, including education, emotion regulation skills, self-disclosure, narrative development, and connection to service and purpose were used throughout the group sessions [67].

### 4.3. Program Themes

#### 4.3.1. Resilience

Resilience training programs to address occupational stress have shown small to moderate positive effects [19,68]. More specifically, interventions using mindfulness or CBT techniques appear to enhance measures of resilience among various populations [68]. Although resilience scores did not significantly improve, the scores were above average at baseline [36] and were maintained throughout the study period. Similarly, Pallesen and colleagues [69] found resilience was moderate to high among nurse managers, despite the high prevalence of personal and work-related burnout in the wake of COVID-19. Results of their cross-sectional study suggest that the cause of burnout symptoms cannot be attributed to low individual resilience.

Literature on the need for resilience in the healthcare workforce has primarily focused on individual resilience, that is, the onus is on the individual to withstand and recover from workplace stress [70], regardless of how demanding or dysfunctional the environment. Though bolstering individual resilience is important for stress management and the ability to navigate high-stress work environments [71], it is not sufficient in addressing burnout or severe stress injuries caused by the environment. Furthermore, for a group of high-performing nurse leaders who exhibit high resilience, the focus of the study might shift to other constructs, such as post-traumatic growth and community or team resilience [72]. This was a rationale for integrating and measuring post-traumatic growth in the present study. During the program, participants learned about myths related to resilience, the impact of the work environment on resilience, and strategies for recovery to reinforce existing personal strengths and coping resources through self-reflection, mindful self-care practices, and connection to joy and purpose [21]. Participants also learned about leader resilience behaviors to strengthen teams [22]. The study results support that resilience training is necessary but not sufficient for individual wellbeing.

#### 4.3.2. Insight

The results show a significant improvement in self-reflection and insight among intervention participants at the endpoint, one-month, and three-month timepoints. Insight is foundational in any therapeutic or personal growth work and is positively associated with wellbeing and satisfaction with life and negatively associated with stress variables [73]. This program focused on methods to enhance this skill, specifically using self-reflection practice to develop a deeper self-understanding, along with strategies to increase insight into emotional, relational, and cognitive processes. Core components of CBT, ACT, and mindfulness involve facilitating insight through in-the-moment processing and self-disclosure. An important aspect of self-awareness taught through mindfulness is self-acceptance, which is the observation of one’s inner experiences and motivations from an objective, non-judgmental perspective [31,73].

Self-reflection as a skill for developing greater self-awareness and insight is also foundational for nursing leadership development [74]) and is a key component in various leadership models, including authentic leadership [33]. Self-reflection involves deliberate consideration of experience and is linked to various benefits [75,76]. Self-reflection in nursing practice can decrease stress and anxiety and increase learning, competency, and self-awareness [77].

Through reflection, nurse leaders develop an awareness of thoughts, biases, feelings, assumptions, limitations, strengths, and behaviors. This self-awareness is key to nurse leaders’ ability to integrate intrapersonal experiences (i.e., attitudes, feelings) with knowledge and clinical experience [74]. Enhanced self-awareness contributes to greater insight into reactions to situations and relational dynamics and enhances emotional intelligence, which is associated with positive empowerment processes, as well as positive organizational outcomes [78]. Moreover, self-awareness gained through purposeful self-reflection practices allows one to intentionally respond to stress in a healthy way so as not to ignore signs of distress. Self-examination, emotional honesty, and critical appraisal of situations, reactions, and intrapersonal experiences were important skills gained through this program.

#### 4.3.3. Self-Compassion

While self-compassion scores did not show improvement at the endpoint, there were significant increases at one-month, three-month, and six-month follow-ups when compared to baseline. The lack of significant improvement immediately following the intervention but improved scores in follow-up timepoints might be explained by the time needed to put new skills into practice and an opportunity to observe a difference in how one responds to oneself in times of struggle.

Practicing self-compassion means responding to oneself with kindness instead of harsh judgment, recognizing that suffering and imperfection are a part of being human, and approaching painful emotions with a balanced mindset [79]. To build such a mindset, participants in this program learned skills to effectively cope with unpleasant emotions, identify and combat self-criticism, avoid overidentifying with emotional states, and recognize the impermanence of all emotions [21]. Self-compassion has been identified as a key mechanism in the effectiveness of mindfulness-based interventions such as this program [80]. A substantial body of literature shows that self-compassion can lead to positive outcomes in both clinical and non-clinical populations [81]. Specifically, self-compassion has been linked with positive mental health, which includes decreased psychopathology [82] and increased positive wellbeing [83]. Self-compassion practice can lead to greater self-esteem, emotion regulation, optimism, motivation, and emotional intelligence [81,84], as well as improvements in self-regulation of health behavior [85].

Studies have shown that low self-compassion levels can be considered a vulnerability factor in the development of burnout, and mindfulness and self-compassion can be protective among healthcare workers [86]. Programs that aim to improve self-compassion in healthcare workers show effects on outcomes such as burnout and STS [87], and in nurses specifically, high levels of self-compassion are associated with lower levels of compassion fatigue [88].

Importantly, self-compassion is a source of resilience and strength when facing adversity [80,83,89]. Participants in this program learned about self-compassion through session content and experiential practice activities (e.g., loving–kindness meditation). An attitude of self-compassion can be adopted inside and outside of the workplace to allow an individual to extend patience to oneself during difficult situations outside of one’s control [80], which is also a cognitive skill of empowerment taught during the program (i.e., internal locus of control). Self-compassion can be a valuable resource for healthcare professionals who often value compassion for others while struggling to care for their own needs [90]. For unit-based nurse leaders who give compassion to others as a feature of their job, learning about self-compassion introduced a new perspective and provided relief when considering their limitations in terms of how much they can give. Self-compassion practice can alleviate the negative ramifications of perfectionism and high or unrealistic performance expectations for high-achieving nurse leaders that may be reinforced in a demanding job.

#### 4.3.4. Empowerment

Empowerment among participants improved in all domains of the Psychological Empowerment Instrument–Meaning, Competence, Self-Determination, and Impact. The sustainability of the increased scores was stronger in the Meaning and Competence subdomains compared to the Self-Determination and Impact subdomains. Meaning and competence are likely to be considered from a personal rather than environmental perspective [39,91]. For healthcare workers, the meaningfulness cognition has the most powerful main effect on burnout symptoms beyond the effect of stressors [92]. Alternatively, Self-Determination and Impact are more often considered through a systems lens. Although unit-based leaders hold some positional and influential power, as an individual-level program, it makes sense why this intervention would impact empowerment more from the personal perspective.

The study findings are consistent with literature suggesting that nurse managers’ structural, psychological, and work empowerment tend to be high or moderately high [93]. Both structural and psychological empowerment negatively correlated with burnout and psychological empowerment had a mediating effect on burnout [94]. Furthermore, psychological empowerment is associated with perceived organizational support and managerial self-efficacy and correlated negatively with emotional exhaustion [93]. Nurse manager empowerment has correlated positively with job satisfaction [93,95].

Because psychological empowerment has beneficial effects, organizations can employ different strategies to enhance it [92], including this psychoeducation group program. Theoretically, both attitudinal and behavioral outcomes have been associated with psychological empowerment, including organizational commitment, turnover intentions, satisfaction, and innovation [91], suggesting that feeling empowered contributes to positive performance and positive perceptions of the job.

### 4.4. Perceived Stress, Job Satisfaction, and Self-Efficacy

Although a significant decrease in perceived stress was only seen at the one-month follow-up timepoint, the maintenance of these scores over the other timepoints provides support for these program themes as skill sets that can safeguard the effects of ongoing stressors. The instrument used to measure stress assesses perceived states of stress, which can easily be affected by circumstances changing regularly in one’s work and home environments [41]. It is believed that the subjective interpretation of stressful events impacts the resulting response, suggesting that it is not the event itself but rather the cognitive appraisal of the event that causes distress [41]. In the case of unit-based nurse leaders, they may acknowledge the tremendous stress involved in their work but also recognize their capacity to manage it, which leads to a resilient mindset.

There is a negative association between job stress and job satisfaction [6]. Although there were no significant changes in job satisfaction in this study, participants’ scores were higher than the mean score (3.98) throughout the study period as measured by a general instrument that is not specific to nurse management job roles [42]. Job satisfaction among nurse managers has been linked with individual health and wellbeing, autonomy and structural empowerment, and social support and team relationships [6]. Similarly, participants’ self-efficacy scores were above average at baseline [40] and were maintained throughout the study period, although there were no significant improvements. Van Dyk and colleagues [96] found self-efficacy among frontline nurse managers to be linked with years of experience. The self-efficacy of unit-based nurse leaders can influence how they perceive their skills to build a healthy work environment for their nurses and ensure the quality and safety of patient care [96].

### 4.5. Limitations

In this study, there is the possibility of social desirability bias due to the self-reported nature of the data collected through validated instruments. Respondents may answer questions in a favorable way, such as overreporting a positive outcome such as compassion satisfaction and underreporting a negative outcome such as burnout. Moreover, this healthcare system recently switched its electronic health record (EHR) platform during the study period. The launch of this platform was a considerable undertaking for nurse leaders and potentially affected participants’ experience in the program and their responses to the self-report instruments. The study sample was employed at the same faith-based healthcare system as previous studies on this psychoeducational group program. To ensure the generalizability of results across healthcare systems, future studies will be conducted in other settings.

### 4.6. Implications

With two pilot studies and two randomized controlled trials conducted on the original program for registered nurses and its adaptation for unit-based nurse leaders [22,25], the program is building empirical support. Empirically Supported Interventions (ESIs) are tested under scientific rigor and show consistently positive results in different trials [97,98]. The direct application of ESIs involves integrating interventions that have some evidence for their efficacy and effectiveness for a given population (e.g., direct care nurses, unit-based nurse leaders) or clinical problem (e.g., burnout) into routine care settings [98]. Collaboration between hospital departments, such as research, operational/clinical leadership, and human resources, can allow for the synthesis of program development, research, and operations to reach nurses with targeted, empirically supported interventions. This study also contributes to the substantial evidence base for the use of ACT, CBT, and mindfulness-based approaches in the prevention and treatment of work stress and burnout [28,61,62].

Healthcare organizations can support unit-based nurse leaders by embedding evidence-based programs developed to reduce stress and burnout symptoms and promote their wellbeing. The program curriculum is applicable and adaptable to members of the nursing workforce of various specialties in all settings [21]. Stakeholder engagement from executive and clinical leadership is necessary for the implementation of this psychoeducational group program, and licensed mental health professionals are required to lead the program groups. Drawing upon theoretical and conceptual frameworks from implementation literature, combined with operations knowledge from key organizational stakeholders, the implementation process should involve collaboration with system partners and providers, plans to sustain changes and growth, and fidelity measures tailored to local contexts to ensure program integrity [97,98,99].

## 5. Conclusions

This study adds to evidence that this psychoeducational group program can be an effective intervention for improving and protecting mental wellbeing, specifically among unit-based nurse leaders, which is a population for which there is a dearth of existing knowledge from cross-sectional and intervention studies. The findings inform how the program can enhance indicators of wellbeing and alleviate distress symptoms of nurse managers and assistant nurse managers in terms of post-traumatic growth, self-reflection and insight, self-compassion, psychological empowerment, compassion satisfaction, stress, burnout, and STS. Literature about nurses’ wellbeing and burnout often states the importance of changes in the practice environment. However, few studies have examined the impact of individual-level interventions on unit- and organization-level outcomes. Future research can examine this relationship with the hypothesis that a psychologically healthy nurse leader can facilitate a healthy workplace culture and lead to positive outcomes related to the staff and patient experience. There is an upcoming secondary analysis planned to examine nurse leader retention and program participants’ unit-level performance indicators, such as the National Database of Nursing Quality Indicators (NDNQI) scores.

## Figures and Tables

**Table 1:** Demographic Characteristics of Participants.

Variable	Intervention (n = 39)		Control (n = 38)		Chi-Square/t	*p*-Value
	Count/Mean	%/SD	Count/Mean	%/SD		
Sex					1.722	0.189
Female	32	82.1%	35	92.1%		
Male	7	17.9%	3	7.9%		
Race					2.857	0.582
Asian	2	5.1%	1	2.6%		
Black	5	12.8%	2	5.3%		
Multi	1	2.6%	1	2.6%		
Other	1	2.6%	0	0.0%		
White	30	76.9%	34	89.5%		
Ethnicity					0.502	0.479
Hispanic	5	12.8%	3	7.9%		
Non-Hispanic	34	87.2%	35	92.1%		
Marital Status					7.429	0.115
Single	10	25.6%	3	7.9%		
Married	24	61.5%	29	76.3%		
Separated	2	5.1%	0	0.0%		
Divorced	2	5.1%	3	7.9%		
Partnered Widowed	1 0	2.6% 0.0%	3 0	7.9% 0.0%		
Education					1.232	0.540
Associate’s degree	5	12.8%	4	10.5%		
Bachelor’s degree	26	66.7%	22	57.9%		
Master’s degree	8	20.5%	12	31.6%		
Job Role					0.312	0.577
ANM	25	64.1%	22	57.9%		
NM	14	35.9%	16	42.1%		
Age	43.97	10.60	44.76	9.24	0.348	0.729
Years of Experience	3.92	4.93	5.00	6.19	0.846	0.400

**Table 2:** Within-Group Comparison (Paired Samples T-Test) at Baseline and Endpoint.

Variables	Baseline	Endpoint	Effect Size	t	*p*-Value
PTGI-Total	67.91 (22.70)	69.65 (27.47)	0.07	−0.37	0.712
PTGI-Relating to Others	21.76 (8.03)	22.65 (9.85)	0.10	−0.52	0.610
PTGI-New Possibilities	15.79 (6.37)	15.88 (7.29)	0.01	−0.07	0.942
PTGI-Personal Strength	13.50 (4.31)	14.56 (5.04)	0.23	−1.13	0.266
PTGI-Spiritual Change	5.97 (2.88)	6.21 (3.13)	0.08	−0.45	0.655
PTGI-Appreciation of Life	10.88 (3.06)	10.35 (3.65)	−0.16	0.88	0.388
BRS	3.81 (0.61)	3.79 (0.60)	0.03	0.24	0.815
SRIS-Total	89.88 (14.41)	93.85 (13.40)	0.29	−2.09	0.045
SRIS-Insight	35.82 (7.37)	37.65 (6.05)	0.27	−1.51	0.142
SRIS-Self-Reflection	54.06 (9.86)	56.21 (9.03)	0.23	−1.63	0.112
SCS-SF	3.19 (0.81)	3.35 (0.81)	0.18	−1.72	0.095
PEI-Total	5.41 (0.90)	5.82 (0.66)	0.52	−2.86	0.007
PEI-Meaning	5.82 (0.86)	6.20 (0.72)	0.48	−2.37	0.024
PEI-Competence	5.35 (1.03)	5.97 (0.76)	0.68	−4.90	<0.001
PEI-Self Determination	5.26 (1.02)	5.63 (0.86)	0.39	−2.08	0.045
PEI-Impact	5.22 (1.16)	5.49 (1.17)	0.23	−1.11	0.275
GSE	33.29 (3.91)	33.91 (3.87)	0.16	−1.66	0.107
PSS	16.62 (8.13)	15.38 (7.11)	−0.16	1.36	0.182
ProQOL-CS	40.91 (5.57)	42.82 (6.28)	0.32	−3.07	0.004
ProQOL-Burnout	23.06 (6.45)	21.47 (6.23)	−0.25	2.30	0.028
ProQOL-STS	24.76 (6.46)	23.76 (6.20)	−0.16	1.52	0.138
BIAJS	4.13 (0.78)	4.12 (0.64)	−0.01	0.07	0.942

Posttraumatic Growth Inventory (PTGI); Professional Quality of Life (ProQOL) Scale; Brief Resilience Scale (BRS); Self-Reflection and Insight Scale (SRIS); Self-Compassion Scale-Short Form (SCS-SF); Psychological Empowerment Instrument (PEI); General Self-Efficacy Scale (GSE); Perceived Stress Scale (PSS); Brief Index of Affective Job Satisfaction (BIAJS).

**Table 3:** Within-Group Comparison (Paired Samples T-Test) at the Follow-Up Timepoints.

Variables	1-Month	Effect Size	t	*p*-Value	3-Month	Effect Size	t	*p*-Value	6-Month	Effect Size	t	*p*-Value
PTGI-Total	72.74 (25.06)	0.20	−1.02	0.317	75.81 (23.16)	0.34	−2.72	0.011	72.09 (25.06)	0.17	−0.83	0.413
PTGI-Relating to Others	23.29 (9.02)	0.18	−0.89	0.379	24.19 (8.44)	0.29	−1.77	0.087	23.06 (9.63)	0.15	−0.55	0.585
PTGI-New Possibilities	16.88 (6.30)	0.17	−0.93	0.359	18.06 (6.03)	0.37	−2.95	0.006	17.16 (6.28)	0.22	−1.14	0.265
PTGI-Personal Strength	15.09 (4.65)	0.35	−1.72	0.095	15.63 (4.23)	0.50	−1.36	0.182	14.72 (4.42)	0.28	−1.36	0.183
PTGI-Spiritual Change	6.41 (2.79)	0.16	−0.80	0.428	6.66 (2.73)	0.25	−1.73	0.093	6.25 (3.25)	0.09	−0.57	0.575
PTGI-Appreciation of Life	11.06 (3.87)	0.05	−0.24	0.810	11.28 (3.42)	0.12	−0.82	0.421	10.91 (3.60)	0.01	0.00	1.00
BRS	3.99 (0.65)	0.29	−1.65	0.109	3.91 (0.77)	0.14	−0.89	0.379	3.81 (0.79)	0.00	−0.04	0.969
SRIS-Total	91.65 (16.69)	0.11	−0.82	0.420	91.28 (16.82)	0.09	−1.11	0.277	88.78 (19.85)	−0.06	0.21	0.837
SRIS-Insight	38.09 (5.97)	0.34	−2.11	0.043	37.78 (6.21)	0.29	−2.05	0.048	36.50 (7.44)	0.09	−0.81	0.422
SRIS-Self-Reflection	53.56 (11.92)	−0.05	0.33	0.745	53.50 (13.12)	−0.05	0.10	0.918	52.28 (13.88)	−0.15	0.73	0.468
SCS-SF	3.42 (0.77)	0.29	−2.82	0.008	3.44 (0.72)	0.33	−3.81	0.001	3.48 (0.86)	0.35	−3.06	0.005
PEI-Total	5.85 (0.69)	0.55	−3.84	0.001	5.79 (0.74)	0.46	−2.09	0.045	5.75 (0.97)	0.36	−1.57	0.126
PEI-Meaning	6.11 (0.68)	0.37	−2.29	0.029	6.10 (0.69)	0.36	−1.43	0.161	6.23 (0.92)	0.46	−2.17	0.038
PEI-Competence	5.88 (0.86)	0.56	−4.52	<0.001	5.79 (1.21)	0.39	−2.42	0.022	5.90 (0.90)	0.57	−3.11	0.004
PEI-Self Determination	5.69 (0.85)	0.46	−2.99	0.005	5.78 (0.90)	0.54	−2.48	0.019	5.51 (1.39)	0.21	−0.75	0.457
PEI-Impact	5.73 (0.92)	0.49	−2.91	0.006	5.47 (1.29)	0.20	−0.53	0.597	5.38 (1.43)	0.12	−0.14	0.893
GSE	33.71 (4.13)	0.10	−0.96	0.344	33.69 (3.84)	0.10	−1.37	0.182	33.03 (5.27)	−0.06	0.00	1.00
PSS	14.74 (7.61)	0.24	2.06	0.048	16.16 (8.09)	−0.06	0.42	0.676	16.34 (8.77)	−0.03	0.27	0.786
ProQOL-CS	42.38 (5.63)	0.26	−2.22	0.033	41.63 (7.18)	0.11	−0.95	0.348	41.38 (8.21)	0.07	−0.59	0.562
ProQOL-Burnout	20.97 (5.02)	−0.36	3.10	0.004	22.41 (7.44)	−0.09	0.49	0.627	21.97 (7.75)	−0.15	0.99	0.332
ProQOL-STS	22.91 (6.48)	−0.29	2.24	0.032	23.00 (8.53)	−0.23	1.53	0.136	23.91 (9.03)	−0.11	0.57	0.573
BIAJS	4.20 (0.70)	0.09	−0.65	0.518	3.96 (0.71)	−0.23	1.58	0.125	4.07 (1.06)	−0.06	0.56	0.579

Posttraumatic Growth Inventory (PTGI); Professional Quality of Life (ProQOL) Scale; Brief Resilience Scale (BRS); Self-Reflection and Insight Scale (SRIS); Self-Compassion Scale-Short Form (SCS-SF); Psychological Empowerment Instrument (PEI); General Self-Efficacy Scale (GSE); Perceived Stress Scale (PSS); Brief Index of Affective Job Satisfaction (BIAJS).

**Table 4:** Between-Group Comparison (Repeated-Measures ANOVA).

Variables	Group	Baseline	Endpoint	1-Month	3-Month	F	*p*-Value
PTGI-Total	Intervention	67.91 (22.70)	69.65 (27.47)	72.74 (25.06)	75.81 (23.16)	6.31	0.015
	Control	59.76 (25.16)	56.09 (25.28)	56.70 (21.66)	57.17 (23.29)		
PTGI-Relating to Others	Intervention	21.76 (8.03)	22.65 (9.85)	23.29 (9.02)	24.19 (8.44)	5.94	0.018
	Control	18.61 (9.87)	17.35 (9.66)	17.36 (8.62)	17.73 (8.12)		
PTGI-New Possibilities	Intervention	15.79 (6.37)	15.88 (7.29)	16.88 (6.30)	18.06 (6.03)	6.41	0.014
	Control	13.89 (6.63)	13.12 (6.27)	12.33 (6.22)	13.13 (6.29)		
PTGI-Personal Strength	Intervention	13.50 (4.31)	14.56 (5.04)	15.09 (4.65)	15.63 (4.23)	4.32	0.042
	Control	12.32 (5.10)	11.94 (5.49)	12.55 (4.37)	12.43 (4.98)		
PTGI-Spiritual Change	Intervention	5.97 (2.88)	6.21 (3.13)	6.41 (2.79)	6.66 (2.73)	6.83	0.011
	Control	5.00 (3.01)	4.35 (2.80)	4.39 (2.84)	4.70 (2.78)		
PTGI-Appreciation of Life	Intervention	10.88 (3.06)	10.35 (3.65)	11.06 (3.87)	11.28 (3.42)	2.69	0.106
	Control	9.95 (3.79)	9.32 (3.55)	10.06 (3.38)	9.17 (3.86)		
BRS	Intervention	3.81 (0.61)	3.79 (0.60)	3.99 (0.65)	3.91 (0.77)	0.015	0.903
	Control	3.75 (0.63)	3.78 (0.65)	3.83 (0.53)	3.90 (0.46)		
SRIS-Total	Intervention	89.88 (14.41)	93.85 (13.40)	91.65 (16.69)	91.28 (16.82)	0.18	0.673
	Control	94.10 (15.01)	92.03 (14.06)	91.58 (13.70)	91.37 (15.09)		
SRIS-Insight	Intervention	35.82 (7.37)	37.65 (6.05)	38.09 (5.97)	37.78 (6.21)	0.11	0.742
	Control	37.71 (6.81)	35.82 (7.64)	35.94 (7.36)	36.10 (7.74)		
SRIS-Self-Reflection	Intervention	54.06 (9.86)	56.21 (9.03)	53.56 (11.92)	53.50 (13.12)	0.63	0.432
	Control	56.39 (10.74)	56.21 (10.69)	55.64 (10.63)	55.27 (10.74)		
SCS-SF	Intervention	3.19 (0.81)	3.35 (0.81)	3.42 (0.77)	3.44 (0.72)	0.08	0.778
	Control	3.18 (0.79)	3.16 (0.72)	3.26 (0.85)	3.30 (0.71)		
PEI-Total	Intervention	5.41 (0.90)	5.82 (0.66)	5.85 (0.69)	5.79 (0.74)	0.45	0.505
	Control	5.55 (0.72)	5.47 (0.99)	5.54 (0.89)	5.64 (0.81)		
PEI-Meaning	Intervention	5.82 (0.86)	6.20 (0.72)	6.11 (0.68)	6.10 (0.69)	1.35	0.250
	Control	5.85 (0.72)	5.72 (1.04)	5.81 (0.90)	5.91 (0.90)		
PEI-Competence	Intervention	5.35 (1.03)	5.97 (0.76)	5.88 (0.86)	5.79 (1.21)	0.01	0.910
	Control	5.75 (0.76)	5.70 (0.99)	5.81 (0.93)	5.69 (0.68)		
PEI-Self Determination	Intervention	5.26 (1.02)	5.63 (0.86)	5.69 (0.85)	5.78 (0.90)	0.20	0.656
	Control	5.34 (1.10)	5.27 (1.15)	5.42 (1.11)	5.59 (0.99)		
PEI-Impact	Intervention	5.22 (1.16)	5.49 (1.17)	5.73 (0.92)	5.47 (1.29)	0.36	0.551
	Control	5.25 (0.98)	5.19 (1.36)	5.10 (1.36)	5.39 (1.29)		
GSE	Intervention	33.29 (3.91)	33.91 (3.87)	33.71 (4.13)	33.69 (3.84)	0.12	0.727
	Control	33.16 (3.66)	32.74 (3.34)	32.79 (3.72)	33.83 (3.59)		
PSS	Intervention	16.62 (8.13)	15.38 (7.11)	14.74 (7.61)	16.16 (8.09)	0.59	0.446
	Control	17.66 (7.47)	17.91 (6.13)	17.42 (6.49)	17.13 (6.90)		
ProQOL-CS	Intervention	40.91 (5.57)	42.82 (6.28)	42.38 (5.63)	41.63 (7.18)	0.95	0.334
	Control	40.66 (5.45)	40.41 (5.64)	40.30 (5.41)	39.90 (6.51)		
ProQOL-Burnout	Intervention	23.06 (6.45)	21.47 (6.23)	20.97 (5.02)	22.41 (7.44)	1.06	0.308
	Control	24.11 (5.86)	23.91 (6.37)	23.97 (6.69)	23.87 (6.20)		
ProQOL-STS	Intervention	24.76 (6.46)	23.76 (6.20)	22.91 (6.48)	23.00 (8.53)	0.15	0.703
	Control	24.05 (6.79)	23.62 (5.63)	23.48 (6.20)	23.33 (6.00)		
BIAJS	Intervention	4.13 (0.78)	4.12 (0.64)	4.20 (0.70)	3.96 (0.71)	0.87	0.354
	Control	3.90 (0.81)	3.89 (0.90)	3.88 (1.02)	3.92 (0.95)		

## Data Availability

The datasets generated and/or analyzed during the current study are not publicly available to protect the privacy of the participants but are available from the corresponding author upon reasonable request.
